# Predictive factors of epidural blood patch success in intracranial hypotension syndrome

**DOI:** 10.1111/head.70191

**Published:** 2026-07-15

**Authors:** Anna Le Moal‐Baczynski, Alexandre Castelli, Vincent Doat‐Sarfati, Sylvain Redon, Anne Donnet, Nicolas Bruder, François Antonini, Jules Henri, Jean‐François Hak, Victor Lebreton, Jeanne Morel, Mehdi Assal, Lionel Velly, Pierre Simeone

**Affiliations:** ^1^ Department of Anesthesiology and Critical Care Medicine Aix Marseille University, AP‐HM, University Hospital Timone Marseille France; ^2^ Department of Anesthesiology and Critical Care Medicine Sorbonne Université, AP‐HP, Pitié‐Salpêtrière Hospital Paris France; ^3^ Paris Brain Institute, Laboratoire GenoVasc, INSERM U1127 Paris France; ^4^ Department of Evaluation and Treatment of Pain Aix Marseille University, AP‐HM, University Hospital Timone Marseille France; ^5^ Department of Anesthesiology and Critical Care Medicine Aix Marseille University, AP‐HM, University Hospital Conception Marseille France; ^6^ Department of Anesthesiology and Critical Care Medicine Aix Marseille University, AP‐HM, North University Hospital Marseille France; ^7^ Neuroimagery Department Aix Marseille University, AP‐HM, University Hospital Timone Marseille France; ^8^ Timone Neuroscience Institute, CNRS Aix Marseille University Marseille France

**Keywords:** cerebrospinal fluid leak, epidural blood patch, orthostatic headache, predictive factors, spontaneous intracranial hypotension

## Abstract

**Objective:**

This study aimed to identify predictors of epidural blood patch (EBP) success in patients with intracranial hypotension syndrome.

**Background:**

The epidural blood patch remains the gold standard treatment for intracranial hypotension syndrome, yet its effectiveness varies, and predictors of sustained success remain uncertain.

**Methods:**

We performed a single‐center retrospective cohort study. We analyzed 139 epidural blood patches performed for non‐obstetric intracranial hypotension syndrome between April 2015 and July 2025. Demographic, clinical, biological, radiological, and procedural data were collected. Complete symptom resolution or highly significant improvement at 1 month was defined as treatment success.

**Results:**

Among 93 patients, 1‐month complete success was achieved after 31% of procedures (43/139). Early improvement within 48 h was associated with higher odds of 1‐month EBP success (adjusted odds ratio [aOR] = 9.70, 95% confidence interval [CI]: 3.66–28.96; *p* < 0.001) as was the occurrence of rebound headache (aOR = 5.34, 95% CI: 1.27–26.44; *p* = 0.028). Conversely, symptom exacerbation during the Valsalva maneuver was associated with lower odds of 1‐month EBP success (aOR = 0.30, 95% CI: 0.10–0.81; *p* = 0.020). Lower baseline platelet and fibrinogen levels and osteophytic leaks were negatively associated with early effectiveness but not with 1‐month outcome. Targeted epidural blood patches and shorter delay from symptom onset to procedure were associated with higher success rates in univariate analysis, though not independently. Model performance was robust with an area under the curve (AUC) of 0.88 (95% CI: 0.80–0.90). No infectious complications were observed; rebound headaches and transient back pain were the most common secondary events.

**Conclusion:**

Early clinical improvement and rebound headache are strong positive predictors of durable epidural blood patch effectiveness in intracranial hypotension syndrome, whereas Valsalva‐related symptom worsening indicates a higher risk of failure. Radiological severity did not predict outcomes in our study. These easily identifiable clinical factors can assist in individualized management and reduce unnecessary repeat procedures.

## Plain Language Summary

Reliable predictors of epidural blood patch effectiveness in intracranial hypotension syndrome are currently lacking. In an analysis of 139 procedures, we found that early clinical improvement and rebound headache strongly predicted complete treatment success at 1 month, while symptom worsening during the Valsalva maneuver predicted failure. These readily accessible clinical markers may help optimize patient selection and guide follow‐up strategies after epidural blood patch treatment.

**Abbreviations**
aOR: adjusted odds ratios
aPTT: activated partial thromboplastin time
AUC: area under the curve
CERAR: Committee for Research in Anesthesiology and Intensive Care
CI: confidence interval
CSF: cerebrospinal fluid
CT: computed tomography
DPO: Data Protection Officer
EBP: epidural blood patch
GDPR: General Data Protection Regulation
ICHD‐3: International Classification of Headache Disorders, 3rd edition
IHS: intracranial hypotension syndrome
IQR: interquartile range
MRI: magnetic resonance imaging
NPV: negative predictive value
NRS: Numeric Rating Scale
PPV: positive predictive value
PT: prothrombin time
ROC: receiver operating characteristic
SD: standard deviation
SLEC: spinal longitudinal extradural CSF collection
STROBE: Strengthening the Reporting of OBservational studies in Epidemiology

## INTRODUCTION

Cerebrospinal fluid (CSF) is a key component of the intracranial compartment, whose overall volume remains constant according to the Monro–Kellie doctrine. Under physiological conditions, CSF pressure reflects intracranial pressure, typically ranging from 5 to 15 mmHg in the supine position. Any disturbance in CSF production, circulation, or reabsorption can disrupt this equilibrium, leading to intracranial hypertension or hypotension due to CSF hypovolemia, which is the underlying mechanism of spontaneous intracranial hypotension or intracranial hypotension syndrome (IHS).

IHS remains underdiagnosed^1^, ^2^ despite advances in imaging and diagnostic tools. According to the third edition of the International Classification of Headache Disorders (ICHD‐3),^3^ IHS is classified under “Headache attributed to low CSF pressure,” and diagnosis relies on orthostatic headache associated with a post‐puncture situation or either a CSF opening pressure < 60 mm H_2_O or imaging related to a CSF leak. In addition, the Bern score improves likelihood of diagnosing intracranial hypotension.^4^

IHS may be secondary (iatrogenic or post‐traumatic) or primary.^5^ Clinically, IHS presents with orthostatic headaches often accompanied by nausea, tinnitus, neck pain, photophobia, diplopia, and occasionally focal neurological deficits. Complications such as subdural hematomas, venous sinus thrombosis, seizures, or even brainstem herniation have been reported, highlighting the need for early recognition and appropriate management.^6^, ^7^, ^8^, ^9^, ^10^

Conservative treatment provides limited effectiveness, with fewer than one‐third of patients achieving full recovery.^11^ The epidural blood patch (EBP), introduced in the 1960s, remains the cornerstone of therapy. Its mechanism combines an immediate effect from transient intracranial pressure elevation and a delayed effect from sealing the dural defect through clot formation, localized inflammatory response,^12^ and elevation of epidural pressure counteracting CSF leakage.^13^ Reported success rates vary widely in the literature (approximately from 30 to 80%), depending on the underlying etiology, whether the EBP is targeted, the timing post‐EBP, and the diagnostic criteria used to define treatment success.^11^, ^14^

Despite its widespread use, EBP effectiveness remains unpredictable,^15^ and most available data derive from obstetric patients, whose physiological characteristics differ from those of the general population. Previous studies attempting to identify predictors of EBP success have been inconsistent and underpowered.^11^, ^16^

We hypothesized that specific clinical, radiological, and procedural factors would be independently associated with the rate of complete therapeutic success at 1 month following an EBP in patients with non‐obstetric intracranial hypotension syndrome. The aim of this study was therefore to identify demographic, clinical, biological, radiological, and procedural factors associated with successful EBP in patients with non‐obstetric IHS.

## METHODS

### Study design and setting

This was a single‐center retrospective cohort study conducted in the Department of Neuroanesthesia and Critical Care at La Timone University Hospital (AP–HM, Marseille, France). The inclusion period extended from April 2015 to July 2025.

### Eligibility criteria

Adults (≥18 years) with primary or secondary IHS and no contraindication to EBP (such as CSF overdrainage due to shunt systems) were eligible. Obstetric cases and individuals under guardianship were excluded.

### Patient selection and diagnostic criteria

Diagnosis of IHS was based on new‐onset orthostatic headache associated with compatible clinical context or imaging findings and confirmed by a specialist physician. Intrathecal pressure measurement was not routinely performed due to its invasiveness and limited diagnostic value when normal.

EBP was indicated for patients with disabling or refractory symptoms despite conservative treatment and associated with impaired quality of life. Treatment effectiveness was assessed through standard follow‐up by consultations.

Some patients received multiple EBPs; therefore, the potential intra‐patient correlation between procedures was considered when interpreting the results.

### Objectives and outcomes

The primary objective was to identify demographic, clinical, biological, radiological, and procedural predictors of EBP success in patients with non‐obstetric IHS. The primary outcome was complete success 1 month after EBP. A secondary outcome was complete success evaluated at 48 h post‐procedure. Complete success was defined as total disappearance of symptoms or marked improvement without need for further intervention. Partial success referred to moderate improvement deemed insufficient by the patient. Failure was defined as either partial success or complete absence of clinical improvement.

### EBP procedure

Patients were treated either as outpatients within the Pain Management Unit or as inpatients in the emergency department or specialized medical units (neurology, internal medicine, or neurosurgery). EBPs were performed by an anesthesiologist in the recovery room or interventional neuroradiology suite under strict aseptic conditions.

Continuous monitoring and intravenous access were maintained. An 18‐gauge Tuohy needle was used, and the epidural space was identified by the loss‐of‐resistance technique using saline. Once located, autologous blood was drawn by a nurse and immediately injected by the operator under sterile conditions. The injected volume was left to the operator's discretion.

Technical parameters—puncture level, injected volume, number of levels (single versus multilevel), patient position, and intra‐procedural symptoms—were recorded. Reported symptoms included local pain, lumbar tension, radicular pain, or transient headache during injection. Post‐procedure, patients were monitored for rebound headache, radiculalgia, back pain, or signs of intracranial hypertension. Pain intensity (Numeric Rating Scale [NRS]) and duration of bed rest were documented. Post‐procedural rebound headache was defined as a transient, non‐orthostatic headache occurring within hours to days after the EBP, typically worsening in the supine position and resolving spontaneously within a few days. In contrast, signs of intracranial hypertension were defined as a persistent, non‐orthostatic headache occurring after the procedure, differing from the initial presentation and not resolving spontaneously, and potentially associated with additional symptoms such as nausea, vomiting, diplopia, or focal neurological deficits. When available, the diagnosis was supported by paraclinical findings (fundoscopy or neuroimaging) or by clinical improvement under acetazolamide treatment.

### Ethical considerations

The study was approved by the Ethics Committee for Research in Anesthesiology and Intensive Care (CERAR; IRB 00010254–2024–088) and by the institutional Data Protection Officer (AP–HM DPO). It complied with the General Data Protection Regulation (GDPR) and did not alter standard patient management. The requirement for written informed consent was waived by the ethics committee due to the retrospective nature of the study, and non‐opposition to data use for research was systematically verified before inclusion.

### Data collection

Clinical data were extracted from electronic medical records, collected retrospectively and prospectively. All assessments were performed by physicians experienced in IHS management.

### Patient characteristics

Demographic data (age, sex, height, weight), medical and surgical history, trauma, and current treatments were recorded. Headache location, orthostatic triggering, worsening with Valsalva maneuver before procedure, and associated symptoms (nausea, vomiting, vertigo, tinnitus, neck pain, diplopia, cranial nerve deficits, focal sensorimotor, cognitive, or balance disturbances) were systematically assessed.

### Etiology and type of leak

Etiology (primary or secondary) and specific type (Type I–IV, post‐lumbar puncture, surgical, or traumatic) were documented.

### Paraclinical evaluation

Pre‐procedural platelet count, fibrinogen level, and coagulation profile, including prothrombin time (PT) and activated partial thromboplastin time (aPTT) were recorded.

Brain magnetic resonance imaging (MRI) findings included meningeal enhancement, venous engorgement, subdural collections, and rostrocaudal descent signs (reduced mamillopontine, prepontine, or suprasellar cisterns; tonsillar descent; pituitary enlargement). Measurements were independently obtained by two blinded raters, and the mean value was used for analysis.

Spinal MRI or myelography findings such as the presence of epidural fluid collections anterior and posterior referred to as spinal longitudinal extradural CSF collection (SLEC +) and direct CSF leaks were documented. Localization of the leak was determined either by imaging findings or by clinical context (e.g., post‐traumatic or post‐procedural origin), although MRI is known to have lower sensitivity than computed tomography (CT) myelography for precise leak localization.

Targeted EBPs were defined as procedures performed at the level of a leak that had been formally identified, either radiologically or based on a highly suggestive clinical context.

### Statistical analysis

Data distribution normality was assessed using the Shapiro–Wilk test. Continuous variables are presented as mean ± standard deviation (SD) or as median and interquartile range (IQR) when not normally distributed. Categorical variables are presented as counts and percentages (*n*, %). Group comparisons were performed using the independent‐samples *t*‐test for normally distributed continuous variables and the Mann–Whitney U test for non‐normally distributed variables. Categorical variables were compared using Fisher's exact test.

Univariable logistic regression analyses were first performed to identify factors associated with 1‐month EBP success. Variables considered clinically relevant or showing an association with the outcome in univariable analyses (*p* < 0.20) were entered into a multivariable logistic regression model to identify independent predictors. Adjusted odds ratios (aORs) with 95% confidence intervals (CIs) were reported. For the multivariable logistic regression model, multicollinearity among predictors was examined, and model calibration was assessed using the Hosmer–Lemeshow goodness‐of‐fit test. Receiver operating characteristic (ROC) curve analysis was performed to evaluate the predictive performance of the model. The optimal cutoff value was determined using the Youden index. Discrimination was evaluated using the area under the receiver operating characteristic curve (AUC) with 95% CI reported. Based on this optimal threshold, the corresponding sensitivity, specificity, positive predictive value (PPV), and negative predictive value (NPV) were calculated.

All statistical tests were two‐tailed, and a *p*‐value <0.05 was considered statistically significant. Missing data were not imputed, and analyses were conducted using complete‐case analysis. A total of 131 patients were included in the multivariable analysis, with eight missing observations (6%), assumed to be missing at random. The statistician was blinded to the data, while the clinician performing the procedure and the physician collecting the data were not involved in the statistical analysis. No a priori statistical power calculation was conducted because this study was based on retrospectively collected data. This analysis represents the primary analysis of these data.

All analyses were performed using JMP software, version 16 (SAS Institute, Cary, NC, USA).

## RESULTS

**FIGURE 1:**
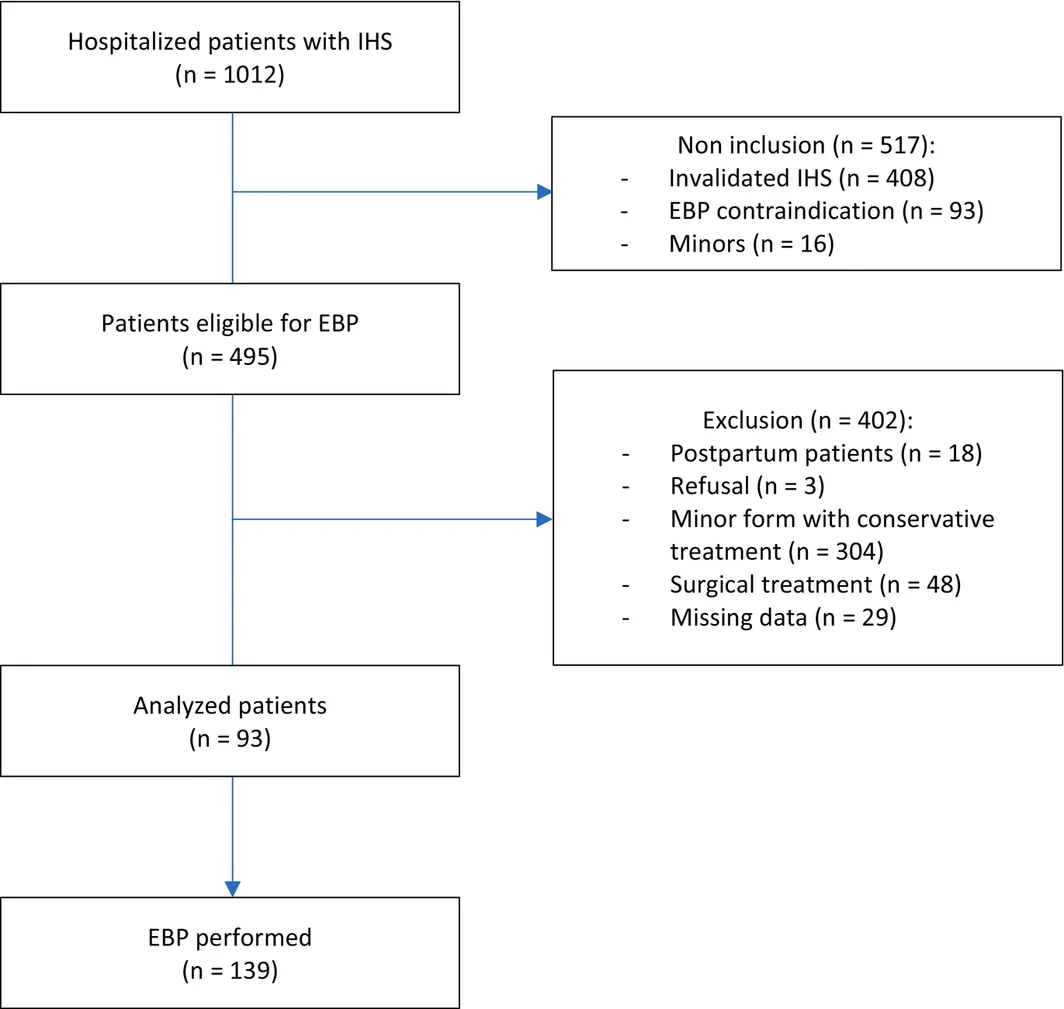
Flow chart. EBP, epidural blood patch; IHS, intracranial hypotension syndrome. [Color figure can be viewed at wileyonlinelibrary.com]

A total of 495 patients were eligible for an EBP; three declined the procedure, 304 were managed conservatively, 48 underwent surgical repair, 18 were obstetrical cases, and 29 were excluded from the final analysis due to incomplete data. Ultimately, 93 patients who received 139 EBPs between April 2015 and July 2025 at La Timone University Hospital (Marseille, France) were included in the study (Figure 1).

Procedural characteristics, along with their univariate comparisons between the “success” and “failure” groups, are summarized in Tables 1 and 2. Radiological characteristics and corresponding univariate analyses are presented in Table 3.

**TABLE 1:** Symptoms and etiology leading to EBP (*n* = 139).

Variable	All procedures (*n* = 139)	Success group (*n* = 43)	Failure group (*n* = 96)	*p*‐Value
Symptoms and signs before the EBP				
Orthostatic headache, *n* (%)	127 (91)	39 (91)	88 (91)	0.800
Nausea/vomiting, *n* (%)	30 (22)	10 (23)	20 (21)	0.191
Worsening during Valsalva maneuver, *n* (%)	61 (44)	11 (26)	50 (52)	**0.003**
Neck stiffness/neck pain, *n* (%)	54 (39)	18 (42)	36 (38)	0.600
Tinnitus, *n* (%)	53 (38)	15 (35)	38 (40)	0.598
Photophobia/phonophobia, *n* (%)	30 (22)	10 (23)	20 (21)	0.922
Dizziness, *n* (%)	46 (33)	15 (35)	31 (32)	0.764
Diplopia, *n* (%)	16 (12)	6 (14)	10 (10)	0.545
Cognitive disorders, *n* (%)	13 (9)	2 (5)	11 (11)	0.344
Sensory or motor deficit, *n* (%)	12 (9)	3 (7)	9 (9)	0.753
Balance disorder, *n* (%)	12 (9)	1 (2)	11 (11)	0.104
Cause of the dural tear				
Primary, *n* (%)	100 (72)	26 (60.5)	74 (77)	**0.043**
CSF venous fistula, *n* (%)	7 (5)	0	7 (7)	**0.021**
Secondary, *n* (%)	38 (27)	17 (40)	21 (22)	**<0.001**
Post‐lumbar puncture, *n* (%)	24 (17)	13 (30)	11 (11)	**0.006**
Post‐traumatic, *n* (%)	9 (6)	0	9 (9)	**0.038**
Dural tear location^a^				
Anterior, *n* (%)	17 (15)	4 (12)	13 (16)	0.774
Posterior, *n* (%)	29 (26)	15 (44)	14 (18)	**0.003**

*Note*: Results are expressed as mean ± standard deviation, median and interquartile range [IQR], or proportions (*n* %). Percentages presented in the descriptive tables are calculated relative to the size of each group (success vs. failure). Bold values indicate statistically significant *p*‐Values.

Abbreviation: EBP, epidural blood patch.

^a^ Data available for 113 EBP.

**TABLE 2:** Peri‐ and post‐procedural characteristics according to blood patch success.

Variable	All procedures (*n* = 139)	Success group (*n* = 43)	Failure group (*n* = 96)	*p*‐Value
Total and adjusted volume				
Volume (mL), median [IQR]	26 [20–33]	27 [20–35]	25 [20–32]	0.396
Volume adjusted for BSA (mL/m^2^), mean (SD)	14.9 ± 4.6	15.1 ± 4.7	14.7 ± 4.6	0.768
Volume adjusted for heigh (mL/m), mean (SD)	15.7 ± 5.2	16.1 ± 5.2	15.6 ± 5.2	0.740
EBP number				
1 EBP, *n* (%)	83 (60)	30 (70)	53 (55)	0.425
2 EBP, *n* (%)	39 (28)	8 (19)	31 (32)	0.425
≥3 EBP, *n* (%)	17 (12)	5 (12)	12 (13)	0.425
Effectiveness at 48 h				
Clinical effectiveness at 48 h, *n* (%)	67 (48)	36 (84)	31 (32)	**<0.001**
Time between symptoms and EBP				
Time (month), median [IQR]	3 [1.1–11.2]	1.6 [0.5–2.5]	4.4 [1.6–13.4]	**<0.001**
Pain scale (NRS)				
NRS ≥7 before EBP, *n* (%)	39 (40)	15 (48)	24 (36)	0.260
NRS ≤3 after EBP, *n* (%)	74 (76)	26 (87)	48 (72)	0.108
Position				
Sitting, *n* (%)	121 (92)	40 (93)	81 (92)	0.843
Lying, *n* (%)	10 (8)	3 (7)	7 (8)	>0.999
Injection site				
Thoracic, *n* (%)	18 (13)	5 (12)	13 (14)	0.792
Lumbar, *n* (%)	122 (89)	37 (86)	85 (90)	0.446
Multilevel, *n* (%)	4 (3)	0	4 (4)	0.310
Procedure conditions				
Targeted EPB, *n* (%)	31 (23)	15 (35)	16 (17)	**0.020**
Fluoroscopy‐guided EBP	5 (4)	2 (5)	3 (3)	0.644
Criterion for stopping the EPB				
Sensation of pressure, *n* (%)	48 (40)	15 (39)	33 (40)	0.976
Pain during injection/radicular pain, *n* (%)	32 (26)	5 (13)	27 (33)	**0.024**
Satisfactory volume, *n* (%)	32 (26)	14 (37)	18 (23)	0.079
Headache, *n* (%)	23 (19)	8 (21)	15 (18)	0.698
Vasovagal episode, *n* (%)	8 (7)	2 (5)	6 (7)	>0.999
EBP side effects				
Back pain, *n* (%)	24 (18)	13 (30)	11 (12)	**0.008**
Rebound headache, *n* (%)	17 (13)	10 (24)	7 (8)	**0.008**
Radicular pain, *n* (%)	4 (3)	1 (2)	3 (3)	>0.999
Intracranial hypertension, *n* (%)	2 (1)	2 (5)	0	0.094

*Note*: Sample sizes vary depending on data availability for each variable. Results are expressed as mean ± standard deviation, median and interquartile range [IQR], or proportions (*n* %). Percentages presented in the descriptive tables are calculated relative to the size of each group (success vs. failure and according to data availability). The different variables are not mutually exclusive. Bold values indicate statistically significant *p*‐Values.

Abbreviations: BSA, body surface area; EBP, epidural blood patch; IQR, interquartile range; NRS, Numeric Rating Scale.

**TABLE 3:** Imaging data characteristics according to blood patch success.

Variable	Global population	Success group	Failure group	*p*‐Value
Bern score, median [IQR]	4 [2–6]	4 [2–6]	4 [2–7]	0.472
Brain MRI (*n* = 103)				
Pachymeningeal enhancement, *n* (%)	57 (53)	16 (53)	41 (52)	0.942
Venous engorgement, *n* (%)	34 (32)	10 (33)	24 (31)	0.829
Pituitary gland enlargement, *n* (%)	31 (29)	5 (17)	26 (34)	0.079
Subdural hematoma, *n* (%)	26 (24)	8 (27)	18 (23)	0.671
Cerebral tonsillar descent, *n* (%)	36 (33)	6 (20)	30 (38)	0.068
Cerebral venous thrombosis, *n* (%)	2 (2)	0 (0)	2 (3)	0.376
Specific measurements of craniocaudal descent (*n* = 76)				
Mamillopontine distance ≤6.5 mm, *n* (%)	45 (63)	12 (60)	33 (63)	0.785
Prepontine cistern ≤5 mm, *n* (%)	43 (60)	10 (50)	33 (63)	0.296
Suprasellar cistern ≤4 mm, *n* (%)	31 (41)	5 (24)	26 (47)	0.062
Mamillopontine distance, mean (SD)	5.9 ± 1.7	6.0 ± 1.3	5.9 ± 1.9	0.746
Prepontine cistern, mean (SD)	4.7 ± 1.5	5.1 ± 1.1	4.2 ± 1.6	0.078
Suprasellar cistern, mean (SD)	4.7 ± 2.2	5.4 ± 1.9	4.5 ± 2.2	0.091
Spinal imaging (*n* = 91)				
Cervical leak, *n* (%)	20 (22)	4 (16)	16 (24)	0.572
Thoracic leak, *n* (%)	27 (30)	8 (32)	19 (29)	0.764
Lumbar leak, *n* (%)	14 (15)	5 (20)	9 (14)	0.452
Anterior epidural collection, *n* (%)	22 (24)	7 (28)	15 (23)	0.275
Posterior epidural collection, *n* (%)	21 (23)	5 (20)	16 (24)	0.668
Length of anterior epidural collection, median [IQR] (number of vertebrae)	6 [4–10]	3.5 [2–6.5]	5 [4.5–12]	0.185
Length of posterior epidural collection, median [IQR]	9.5 [7–12.2]	9 [8.5–10.5]	9 [7–12]	0.875

*Note*: Sample sizes vary depending on the available data for each examination. Results are expressed as mean ± standard deviation, median and interquartile range [IQR], or proportions (*n* %). Percentages presented in the descriptive tables are calculated relative to the size of each group (success vs. failure) and data availability.

Abbreviation: IQR, interquartile range; MRI, magnetic resonance imaging.

### Demographic and univariate analyses

The mean age of the cohort was 44.1 ± 15.1 years, with a female predominance (59/93 patients, 63%). Seventeen patients (17/93, 18%) had a history of chronic headache, 15 (15/93, 16%) experienced chronic pain, and 20 (20/93, 21%) had anxiety or depression. Demographic characteristics, comorbidities (Supplementary Table S1 and Table 1), analgesic management, and coagulation profiles did not differ significantly between success and failure groups.

A new‐onset orthostatic headache was reported before 127/139 (91%) procedures. Symptom exacerbation during Valsalva maneuver occurred more frequently in the failure group (50/96 [52%] vs. 11/43 [26%]; *p* = 0.003). Other presenting symptoms were comparable between groups.

Of the 139 EBP performed for intracranial hypotension cases, 100/139 (72%) were classified as primary and 38/139 (27%) as secondary; one unclassified case was initially presumed primary but later attributed to lumbar puncture. Primary etiology was more frequent in the failure group (74/96 [77%] vs. 26/43 [60%]; *p* = 0.043), whereas secondary causes were more often associated with success (17/43 [40%] vs. 21/96 [22%]; *p* < 0.001). Effectiveness did not differ between primary subtypes I and II. In contrast, all CSF venous fistulas (7/96 [7%] vs. 0; *p* = 0.021) and traumatic leaks (9/96 [9%] vs. 0; *p* = 0.038) occurred in the failure group.

### Imaging findings

Leak localization, determined from imaging or clinical context, was available among 113 procedures. It was anterior in 17/113 cases (15%) and posterior in 29/113 (26%). Posterior leaks were more frequent in the success group (15/34 [44%] vs. 14/79 [18%]; *p* = 0.003). All results are summarized in Table 3.

Brain MRI was performed in 103/139 cases (74%), with 76/139 (55%) available for quantitative craniocaudal measurements. Imaging features consistent with CSF leak did not differ significantly between groups.

The median Bern score was 4 [IQR 2–6] (success: 4 [IQR 2–6] vs. failure: 4 [IQR 2–7]; *p* = 0.472). Among epidural collections, thoracic leaks were most frequent (27/91 [30%]), followed by cervical (20/91 [22%]) and lumbar (14/91 [15%]) sites. The mean cranio‐caudal extent of posterior epidural detachment was 9.5 [IQR 7–12.2] vertebral levels versus 6 [IQR 4–10] for anterior collections.

### Procedural description and associated clinical features

Overall, 43/139 (31%) procedures achieved complete effectiveness at 1 month, while 96/139 (69%) were ineffective. Among the latter, 41/139 (29%) resulted in partial improvement and 55/139 (40%) in no improvement. Effectiveness over time is described in Table S2.

Previous EBPs, injected volume (even when normalized for height or body surface area), and injection level did not differ between groups. Early improvement within 48 h was strongly associated with 1‐month success (36/43 [84%] vs. 31/96 [32%]; *p* < 0.001) (Table 2). The median time from symptom onset to procedure was shorter in the success group (1.6 [IQR 0.5–2.5] vs. 4.4 [IQR 1.6–13.4] months; p < 0.001).

Most EBPs were performed in the sitting position (121/131 [92%]), predominantly at the lumbar level (122/137 [89%]); one in four procedures was targeted (31/137 [23%]). Targeted EBPs were more frequently associated with success (15/43 [35%] vs. 16/94 [17%]; *p* = 0.020).

Procedures interrupted due to pain or radiculalgia occurred more often in the failure group (27/83 [33%] vs. 5/38 [13%]; *p* = 0.024). Post‐procedural back pain was more frequent among responders (13/43 [30%] vs. 11/96 [12%]; *p* = 0.008), as were rebound headaches (10/42 [24%] vs. 7/93 [8%]; p = 0.008).

### Secondary analysis: 48‐h outcomes

Factors associated with early effectiveness at 48 h largely overlapped with those linked to 1‐month outcomes. Valsalva‐induced symptom worsening remained associated with early failure (38/67 [57%] vs. 23/72 [32%]; *p* = 0.028), while posterior leaks were more frequent in the early success group (20/29 [69%] vs. 9/29 [31%]; *p* = 0.004) (data not shown).

Additional factors were identified at 48 h but not at 1 month, including osteophytic (type I) leaks (13 [81%] vs. 3 [19%]; *p* = 0.015), lower platelet count (266.5 ± 59.3 vs. 291.1 ± 68.1 G/L; *p* = 0.025), and lower fibrinogen levels (2.9 ± 0.5 vs. 3.3 ± 0.7 g/L; *p* = 0.011) (Table S3).

### Multivariable analysis

A multivariable logistic regression model was constructed including six variables selected based on clinical relevance: time to EBP, targeted approach, post‐traumatic etiology, early improvement within 48 h, Valsalva‐related symptom exacerbation, and rebound headache occurrence. After adjustment for these variables, two factors remained independently associated with 1‐month EBP success: early improvement within 48 h (aOR = 9.70, 95% CI: 3.66–28.96; *p* < 0.001) and rebound headache occurrence (aOR = 5.34, 95% CI: 1.27–26.44; *p* = 0.028), both associated with higher odds of success. Valsalva‐related symptom exacerbation was associated with lower odds of success (aOR = 0.30, 95% CI: 0.10–0.81; *p* = 0.020). Data are shown in Table S4.

Time to EBP, targeted procedure, and post‐traumatic causes did not remain significant.

**FIGURE 2:**
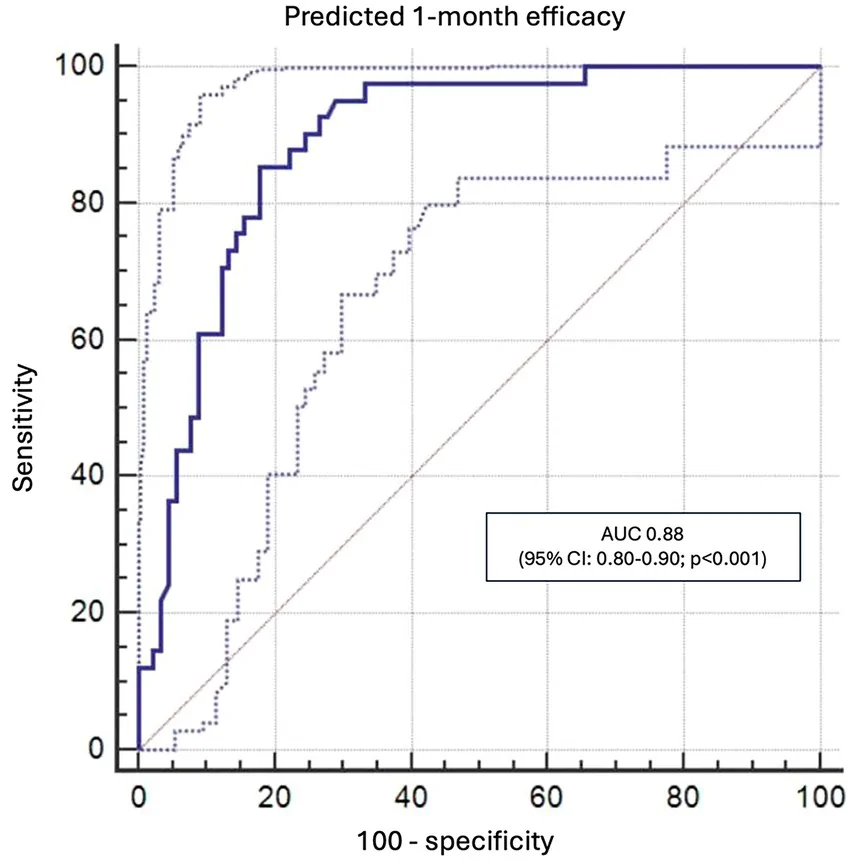
ROC curve for the multivariable model. AUC, area under the curve; ROC, receiver operating characteristic. [Color figure can be viewed at wileyonlinelibrary.com]

Model performance was satisfactory, with an area under the ROC curve (AUC) of 0.88 (95% CI: 0.80–0.90; *p* < 0.001) (Figure 2). The optimal cutoff determined by the Youden index (0.67) yielded a sensitivity of 85.3% (95% CI: 70.8–94.4) and a specificity of 82.2% (95% CI: 72.7–89.5), with PPVs and NPVs of 68.6% (95% CI: 54.1–80.9) and 92.5% (95% CI: 84.4–97.2), respectively.

## DISCUSSION

In our cohort, the overall effectiveness rate of the EBP at 1 month was 31%, while 69% were classified as failures, with outcomes varying according to etiology. Several factors remained significant in multivariable analysis: early improvement within 48 h and the occurrence of rebound headache were independent predictors of success, whereas symptom worsening during transient intracranial pressure elevation (e.g., Valsalva maneuver) independently predicted failure. In univariate analysis, primary versus secondary etiology, posterior leak location, post‐traumatic context, CSF venous fistula, targeted injection, shorter delay to procedure, and post‐procedural back pain were also associated with treatment effectiveness, but these associations did not persist in multivariable analysis.

The 48‐h response rate in our cohort was consistent with previous reports and even appeared slightly higher than that described in some obstetric series.^14^ In contrast, the complete success rate at 1 month was 31%, which is lower than that reported in many prior studies. Several factors may explain this difference. First, only a minority of procedures in our cohort were targeted, whereas studies reporting higher success rates often included a greater proportion of targeted EBPs. Second, cross‐study comparisons are limited by substantial heterogeneity in outcome timing, as most series report either earlier assessments (48 h to 14 days) or later assessments (3 to 6 months).^14^, ^17^ Earlier time points may overestimate durable benefit by capturing transient improvement or spontaneous recovery. For this reason, we selected the 1‐month outcome as the primary endpoint to better distinguish early response from sustained clinical benefit. Third, our definition of complete success was more stringent than that used in some previous studies,^18^ which likely contributed to the lower observed rate. Finally, our cohort reflects real‐world tertiary referral practice and included heterogeneous etiologies, notably CSF venous fistulas and post‐traumatic leaks, which are known to respond less favorably to EBP.

Early improvement (<48 h) was strongly associated with long‐term success, suggesting that lack of early benefit could indicate lower likelihood of durable response. While previous studies^18^, ^19^ identified this phenotype as a “good responder” profile without confirming its predictive value, our findings align with those of Ferrante et al.^20^ who demonstrated persistence of early improvement over time. Nevertheless, a subset of our patients exhibited delayed response at 1 month despite no initial improvement, typically in cases with a long interval between symptom onset and EBP (>90 days), possibly reflecting delayed healing or spontaneous closure of the dural defect. Conversely, early improvement in patients treated very soon after symptom onset may reflect natural recovery rather than treatment effect. Hence, lack of early effectiveness should not be interpreted as definitive failure, but rather as a signal warranting timely reassessment of management strategy according to clinical severity, disease duration, and underlying cause.

Rebound headache after EBP was significantly associated with 1‐month success, consistent with the observations of Pagani‐Estevez et al.^21^ Far from being a complication, this phenomenon may reflect reestablishment of normal intracranial pressure and restoration of CSF homeostasis. Several authors have proposed indirect evidence of this effect through transcranial Doppler or optic nerve sheath diameter measurements before and after EBP.^22^, ^23^ Therefore, rebound headache should be regarded as a positive prognostic marker and actively monitored in clinical practice; its occurrence should discourage immediate repetition of the procedure, which could increase the risk of iatrogenic intracranial hypertension. Importantly, rebound headache occurs after the procedure and therefore cannot be used to select patients for an initial EBP. Instead, it should be interpreted as a post‐procedural prognostic marker of treatment response.

Symptom aggravation during Valsalva maneuver performed before the procedure, reflecting transient intracranial pressure elevation, was identified as an independent predictor of failure. This finding likely indicates the presence of a large dural defect sensitive to pressure fluctuations. A similar association was reported by Pagani‐Estevez et al.^21^ and may help identify patients who could benefit from an early or targeted repeat EBP.

All EBPs performed in post‐traumatic contexts were ineffective, although this association did not persist in multivariable analysis, likely due to the small number of traumatic cases. These findings may indicate atypical, high (craniocervical), or complex dural tears for which accurate localization prior to intervention could help guide more targeted, conservative, or surgical management, avoiding unnecessary procedures.

Targeted EBPs were more often successful in univariate analysis but not confirmed as independent predictors. Although several studies have reported superior effectiveness with targeted approaches, results remain controversial.^24^ Cervical or thoracic injections, while potentially effective, are technically demanding and carry greater procedural risk. Moreover, accurate leak localization is not always feasible due to limitations in imaging^25^, ^26^, ^27^ or expertise, restricting the applicability of targeted EBPs in routine practice. Thus, despite encouraging trends, non‐targeted EBP remains a cornerstone of IHS management.

A longer delay between symptom onset and EBP was associated with poorer outcomes in univariate analysis, consistent with previous predictive studies.^28^ Late intervention may allow symptom chronicization and partial scarring of the dural defect, thereby reducing responsiveness to EBP.

Smaller prepontine and suprasellar cistern heights were correlated with treatment failure, in line with reports linking craniocaudal brain sagging to unfavorable prognosis.^17^ Neither the total number of imaging abnormalities (high Bern score) nor the extent of epidural fluid collection was associated with outcome in our study, suggesting that radiological severity does not necessarily predict EBP effectiveness, although this may reflect limited imaging data and sample size.

Unlike studies suggesting higher success rates with larger injection volumes,^11^ we found no correlation between injection large volumes and effectiveness, even after normalization for body size, in line with the findings of Chestnut et al.^29^ Although expert consensus^30^ recommends a minimum of 20 mL and supports the safety of larger volumes, but their benefit remains uncertain. Larger randomized studies, particularly in primary IHS, are needed.

In secondary analysis, non‐responders at 48 h had significantly lower platelet counts and fibrinogen levels. Although within normal ranges, these differences suggest that initial clot formation quality may influence early EBP success. Early failure also predicted 1‐month failure, supporting the hypothesis of the importance of effective initial sealing. These findings are consistent with studies showing the potential benefit of platelet‐ or fibrin‐enriched injections.^31^, ^32^ Similarly, osteoarthritis type I osteophytic leaks were more often associated with early failure, possibly due to limited diffusion of injected blood within degenerated or fibrotic tissue, reinforcing the rationale for targeted approaches in these patients.

No infectious complications were reported. However, we observed several procedure‐related adverse events, including two cases of iatrogenic intracranial hypertension, one prolonged radicular deficit, and one procedural dural puncture, all resolving without sequelae at 1 month. These highlight that, despite a favorable safety profile, EBP remains an invasive procedure requiring careful monitoring.

This study's strengths include a comprehensive and systematic data collection process reproducible in routine practice. Initial clinical assessments were performed by a consistent, experienced team, ensuring standardized evaluation, and minimizing interobserver variability. The parameters analyzed are simple and clinically accessible, supporting direct application of our findings.

Regarding internal validity, our multivariable model—including six variables, three of which remained significant—demonstrated excellent discriminatory performance (AUC = 0.88) and good balance between sensitivity and specificity. The high NPV indicates that the model reliably identifies patients unlikely to benefit from EBP under similar conditions, thus avoiding unnecessary repeat procedures.

In terms of external validity, demographic characteristics of our cohort were comparable to those reported in previous studies. Unlike many series focusing exclusively on targeted or small‐volume EBPs, our study reflects real‐world practice where precise leak localization is often uncertain. The overall consistency and physiopathological plausibility of our results support their credibility, although confirmation in prospective cohorts is warranted.

Several limitations should be acknowledged. First, the retrospective, single‐center design conducted in a tertiary‐care setting introduces potential selection and information bias, as our population likely included more severe or chronic cases. In addition, diagnostic confirmation did not always strictly follow ICHD‐3 criteria, which themselves have limitations, potentially leading to rare misclassifications. The lack of standardized questionnaires may also have resulted in incomplete clinical data, particularly regarding headache characteristics, and no validated quality‐of‐life measures were available.

Second, radiological assessment presents several limitations. Incomplete availability of quantitative brain MRI measurements may have introduced selection bias in the radiological analyses, although the proportion of available measurements was similar between success and failure groups. Radiological abnormalities may persist after an EBP despite clinical improvement, and because follow‐up imaging was not systematically available, delayed radiological evolution could not be assessed. Furthermore, leak localization was not systematically confirmed by CT myelography, the reference standard, and was in some cases inferred from MRI findings or clinical context, which may have introduced misclassification bias and limited the accuracy of anatomical localization.

Third, our cohort included heterogeneous etiologies, including CSF venous fistulas and post‐traumatic leaks, which likely differ in pathophysiology and may respond differently to EBP. As discussed above, this heterogeneity may partly explain the lower success rate observed compared to the literature and may limit the generalizability of our predictive model.

Finally, several methodological limitations related to statistical analysis should be considered. Some predictors exhibited zero‐cell counts (perfect separation), which may compromise estimation in standard multivariable models; penalized methods (e.g., Firth's correction) were not used, and results for these variables should be interpreted cautiously. In addition, the number of events relative to the number of predictors included in the multivariable model was limited, increasing the risk of overfitting despite clinically driven variable selection. The comparison between targeted and non‐targeted EBPs may also be subject to confounding, as targeted procedures are only feasible when a leak is radiologically identified, making this variable a proxy rather than an independent factor. Moreover, multiple univariate analyses were performed without adjustment for multiple comparisons, increasing the risk of type I error and the likelihood of identifying spurious associations. In addition, intra‐patient correlation was not explicitly modeled, which represents a further methodological limitation given that multiple procedures were performed in some patients. Although this may violate the assumption of independence and lead to underestimated standard errors, the impact of clustering is likely limited given that nearly half of patients underwent a single procedure and that procedural heterogeneity was substantial. Furthermore, the relatively low number of outcome events constrained the use of more complex approaches such as generalized estimating equations or mixed‐effects models, which could have led to model instability and overfitting. Therefore, a standard multivariable logistic regression was retained, in line with common practice in predictive studies with limited events‐per‐variable. Nonetheless, future studies with larger sample sizes and more structured repeated measures should incorporate appropriate methods to account for within‐patient correlation. These findings should therefore be interpreted cautiously and considered primarily exploratory.

## CONCLUSION

This study identified several prognostic factors associated with EBP effectiveness in IHS. Early clinical improvement within 48 h was a strong predictor of 1‐month success, whereas symptom exacerbation during Valsalva maneuver independently predicted treatment failure. The occurrence of rebound headache emerged as a positive marker and should not be misinterpreted as a complication. Conversely, the initial severity of radiological findings did not correlate with poorer outcomes and should neither discourage anesthesiologists from performing EBP nor prompt premature surgical referral.

In a context where IHS remains underrecognized, these findings provide simple, clinically applicable indicators to guide patient selection and treatment strategy. Nevertheless, prospective multicenter studies are warranted to confirm these results and refine evidence‐based management of IHS.

## AUTHOR CONTRIBUTIONS

**Anna Le Moal‐Baczynski:** Data curation; writing – original draft; conceptualization; writing – review and editing. **Alexandre Castelli:** Conceptualization; methodology; formal analysis; writing – original draft; writing – review and editing. **Vincent Doat‐Sarfati:** Validation; writing – review and editing. **Sylvain Redon:** Data curation; formal analysis. **Anne Donnet:** Data curation; formal analysis. **Nicolas Bruder:** Writing – review and editing; validation. **François Antonini:** Formal analysis; writing – review and editing. **Jules Henri:** Conceptualization; methodology. **Jean‐François Hak:** Formal analysis. **Victor Lebreton:** Conceptualization; methodology; formal analysis. **Jeanne Morel:** Writing – review and editing. **Mehdi Assal:** Writing – review and editing. **Lionel Velly:** Supervision; validation. **Pierre Simeone:** Conceptualization; methodology; investigation; validation; supervision; visualization; writing – original draft; writing – review and editing.

## FUNDING INFORMATION

None.

## CONFLICT OF INTEREST STATEMENT

**Anna Le Moal‐Baczynski, Alexandre Castelli, Vincent Doat‐Sarfati, Sylvain Redon, Anne Donnet, Nicolas Bruder, François Antonini, Jules Henri, Jean‐François Hak, Victor Lebreton, Jeanne Morel, Mehdi Assal, Lionel Velly**, and **Pierre Simeone** have no conflicts of interest to declare.

## DECLARATIONS

The Strengthening the Reporting of OBservational studies in Epidemiology (STROBE) Checklist was used for this cohort study.

## Supporting information


Table S1.

## Data Availability

Anonymized data are available to qualified investigators on request for the purposes of replicating procedures or results by contacting the corresponding author.
