# Supplementary material for: Predictive factors of epidural blood patch success in intracranial hypotension syndrome
Source: Headache. 2026 Jul 15;66(8):1847–58. doi: 10.1111/head.70191 (PMC13571055; doi:10.1111/head.70191)
Supplement: Supplementary file 1 [file HEAD-66-1847-s001.docx]

**Predictive Factors of Epidural Blood Patch Success in Intracranial Hypotension Syndrome**

***Supplementaries***

***Table of contents***

*Supplementary Table 1. Demographic and clinicacl characteristics of patients*

*Supplementary Table 2. Success and failure over time*

*Supplementary Table 3. Clinical and laboratory characteristics according to 48 hours efficacy*

*Supplementary Table 4. Univariable and multivariable logistic regression analyses*

**Supplementary Table 1.** Demographic and clinical characteristics of patients (n=93).

| **Variable** | **Global population**  **(n = 93)** |
| --- | --- |
| Age (years), mean (SD) | 44.1 ± 15.1 |
| Female sex, n (%) | 59 (63) |
| BMI (kg/m²)†, mean (SD) | 24.6 ± 3.8 |
| Height (cm)†, mean (SD) | 169.7 ± 9.6 |
| BSA (m²)†, mean (SD) | 1.81 ± 0.2 |
| Previous headache, n (%) | 17 (18) |
| Chronic pain, n (%) | 15 (16) |
| Anxiety / depression, n (%) | 20 (22) |

*Results are expressed as mean ± standard deviation, median and interquartile range [IQR], or proportions (n %).*

*BMI: body mass index ; BSA: body surface area.*

*† anthropometric data available for 58 patients*

**Supplementary Table 2. Success and failure over time**

Percentages were calculated based on the total study population.

| **Timing of efficacy assessment** | **Population** |
| --- | --- |
| 48 hours success, n (%) | 67 (48) |
| 48 hours failure, n (%) | 72 (52) |
| 1 month success, n (%) | 43 (31) |
| 1 month failure, n (%) | 96 (69) |
| 48 hours success and 1 month success, n (%) | 36 (26) |
| 48 hours success and 1 month failure, n (%) | 31 (22) |
| 48 hours failure and 1 month success, n (%) | 7 (5) |
| 48 hours failure and 1 month failure, n (%) | 65 (47) |

**Supplementary Table 3.** Clinical and laboratory characteristics according to 48 hours efficacy

| **Variable** | **Global population** | **Success group** | **Failure**  **group** | **p value** |
| --- | --- | --- | --- | --- |
| Osteophyte-related leak (Type I), n (%) | 16 (12) | 3 (19) | 13 (81) | **0.015** |
| Platelet count (G/L), mean (SD) | 278.7 ± 63.1 | 291.1 ± 68.1 | 266.5 ± 59.3 | **0.025** |
| Fibrinogen level (g/L), mean (SD) | 3.2 ± 0.7 | 3.3 ± 0.7 | 2.9 ± 0.5 | **0.011** |
| Valsalva maneuver, n (%) | 61 (43) | 23 (38) | 38 (62) | **0.028** |
| Posterior leak, n (%) | 29 (20) | 20 (69) | 9 (31) | **0.004** |

*Results are expressed as mean ± standard deviation, median and interquartile range [IQR], or proportions (n %). Percentages are calculated within each row.*

**Supplementary Table 4.** Univariable and multivariable logistic regression analyses

| Covariate | Univariate OR | 95% CI | p-value | Multivariate aOR | 95% CI | p-value |
| --- | --- | --- | --- | --- | --- | --- |
| Post-lumbar puncture | 3.35 | [1.36 – 8.42] | 0.014 |  |  |  |
| Worsening during Valsalva maneuver | 0.32 | [0.14 – 0.68] | 0.006 | 0.30 | [0.10 – 0.81] | 0.020 |
| Posterior dural tear | 3.67 | [1.51 – 9.06] | 0.007 |  |  |  |
| Post-traumatic dural tear* | NC |  | 0.057 | NC | NC | 0.992 |
| CSF venous fistula***** | NC |  | 0.110 |  |  |  |
| Primary dural tear | 0.38 | 0.17 - 0.83 | 0.025 |  |  |  |
| Secondary dural tear | 2.83 | [1.28 – 6.34] | 0.016 |  |  |  |
| Targeted EBP | 2.61 | [1.14 – 6.01] | 0.036 | 1.71 | [0.56 – 5.33] | 0.344 |
| Pain during injection / radicular pain | 0.31 | [0.10 – 0.84] | 0.043 |  |  |  |
| Radicular pain after EBP | 3.27 | [1.33 – 8.23] | 0.016 |  |  |  |
| Rebound headache | 3.84 | [1.36 – 11.41] | 0.018 | 5.34 | [1.27 – 26.44] | 0.028 |
| Clinical effectiveness at 48 hours | 10.78 | [4.54 – 28.95] | <0.001 | 9.70 | [3.66 – 28.96] | < 0.001 |
| Mean value of suprasellar cistern | 1.22 | [0.96 – 1.57] | 0.094 |  |  |  |
| Mean value of prepontine cistern | 1.31 | [0.93 – 1.89] | 0.078 |  |  |  |
| Time between symptoms and EBP, in months | 1.00 | [0.99 – 1.00] | < 0.001 | 1.00 | [1.00 – 1.00] | 0.084 |

**Complete separation: no patients in the effective group exhibit this characteristic. The OR cannot be reliably estimated (quasi-separation).*

*Unadjusted ORs derived from simple logistic regressions.*

*Adjusted ORs derived from a multiple logistic regression model including a priori: worsening during Valsalva maneuver, targeted EBP, post-traumatic dural tear, rebound headache, clinical effictiveness at 48 hours, time between symptoms and EBP (n = 131 with complete data).*

*95% CI: 95% confidence interval.*
